# Mesenchymal stem cell-derived extracellular vesicles in therapy against fibrotic diseases

**DOI:** 10.1186/s13287-021-02524-1

**Published:** 2021-08-04

**Authors:** Yuling Huang, Lina Yang

**Affiliations:** Departments of Geriatrics, The First Affiliated Hospital of China Medical University, 155th Nanjing North Street, Shenyang, 110001 Liaoning People’s Republic of China

**Keywords:** Fibrosis, Mesenchymal stem cells, Extracellular vesicles

## Abstract

Fibrosis is likely to occur in many tissues and organs to induce cicatrisation and dysfunction. The therapeutic regimens for delaying and even reversing fibrosis are quite limited at present. In nearly a decade, mesenchymal stem cells (MSCs) have been widely acknowledged as useful in treating fibrotic diseases in preclinical and clinical trials. Further preclinical studies indicated that the effects of mesenchymal stem cell-derived extracellular vesicles (MSC-EVs) are probably superior to that of MSCs. At present, MSC-EVs have attracted much attention in treating fibrosis of lung, liver, kidney, skin, and heart. By contrast, a significant knowledge-gap remains in treating fibrosis of other tissues and organs (including uterus, gastrointestinal tract, and peritoneum) with the aid of MSC-EVs. This review summarises the preclinical research status of MSC-EVs in treating fibrotic diseases and proposes solutions to existing problems, which contribute to further clinical research on the treatment of fibrotic diseases with MSC-EVs in the future.

## Introduction

Fibrosis is regarded as the main cause of disability and death from many diseases. Fibrotic diseases may happen in various main human organs, including idiopathic pulmonary fibrosis (IPF), liver cirrhosis, renal fibrosis and cardiac fibrosis. Research shows that the mortality associated with organ fibrosis reaches nearly 50% in developed countries [1]. The difficulty in reversing fibrosis progress poses a challenge to treatment, which drives the emergence of new therapeutic methods. Numerous preclinical and clinical studies show that mesenchymal stem cells (MSCs) can relieve the progress of fibrosis of various organs [2]. Nevertheless, the mechanism of action underpinning the treatment of fibrotic diseases with mesenchymal stem cell-derived extracellular vesicles (MSC-EVs) remains under exploration. Thus, the review summarises preclinical studies on treating fibrotic diseases with MSC-EVs and proposes solutions to the existing problems.

### MSC-EVs

MSCs, which are classified as multipotential stem cells, are widely available and can be separated from multiple tissues including bone marrow (bone marrow-derived MSCs (BMMSCs)), adipose tissues (adipose-derived MSCs (ADMSCs)), human umbilical cord (huMSCs), human placenta (hpMSCs), human liver (HLSCs), and menstrual blood (MenSCs). Previous studies suggested that MSCs function in target cells through differentiation and paracrine action; however, recent research shows that MSCs act in the treatment of various diseases mainly by excreting extracellular vesicles (EVs) and soluble nutritional factors [3]. At first, transplanted MSCs are directed to lesions through homing [4] and then deliver EVs and nutritional factors based on different methods, including forming tunnelling nanotubes and fusing with cells [5].

**Fig. 1:**
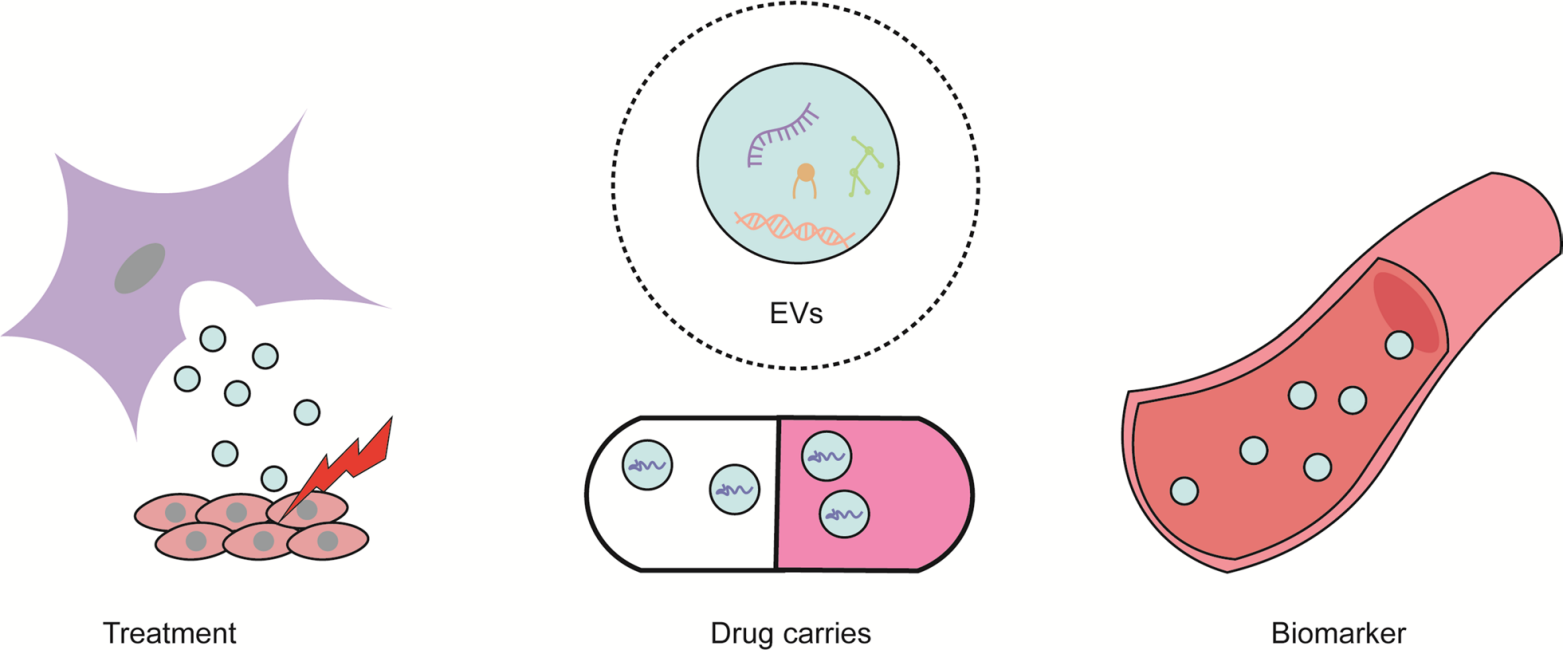
Specific proteins, RNA, DNA, and lipids are packed into EVs. Current research on EVs focuses on three aspects, including EVs as a treatment, a drug carrier, and a biomarker. EVs, extracellular vesicles

EVs can be divided into microvesicles (MVs), exosomes (Exos), and apoptotic bodies according to size, with respective diameters of 100–1000 nm, 30–150 nm, and larger than 1000 nm. Specific proteins, RNA, DNA, and lipids are packed into EVs during sorting [6]. Current research on EVs focuses on three aspects (Fig. 1): firstly, diseases are treated with EVs, for example, miR-150-5p in EVs from ADMSCs (ADMSC-EVs) relieves liver fibrosis by inhibiting the CXCL1 expression [7]. Secondly, EVs serve as a drug carrier: for example, MSC-EVs can be loaded with miR-101a showing an anti-fibrosis effect, thus mediating the cardioprotection with no need for direct intramuscular injection [8]. Thirdly, EVs act as a biomarker: for example, EVs can be used in diagnosis of various cancers and other diseases [9].

### Fibrosis and epithelial mesenchymal transition

Fibrosis is actually a protective reaction required in the repair of damaged cellular tissue; however, fibrosis induces irreversible scarring and reduction in organ function when cellular tissue becomes severely damaged. The main changes of fibrosis include migration and proliferation of fibroblasts and accumulation of extracellular matrices, which damage the organ structure and functions [10]. The epithelial mesenchymal transition (EMT) is associated with the generation and development of fibrosis. EMT corresponds to a biological process during which epithelial cells are transformed into an interstitial cell phenotype through a specific procedure. Thereafter, epithelial cells lose their characteristics and are transformed into mesenchymal cells with loose structures. According to the differences between specific biological environments, EMT can be classified into three types, which are separately closely related to embryogenesis, tissue regeneration, and tumour invasion and metastasis (tissue regeneration forms the focus of the present discussion) [11].

## MSC-EVs and fibrotic diseases

**Fig. 2:**
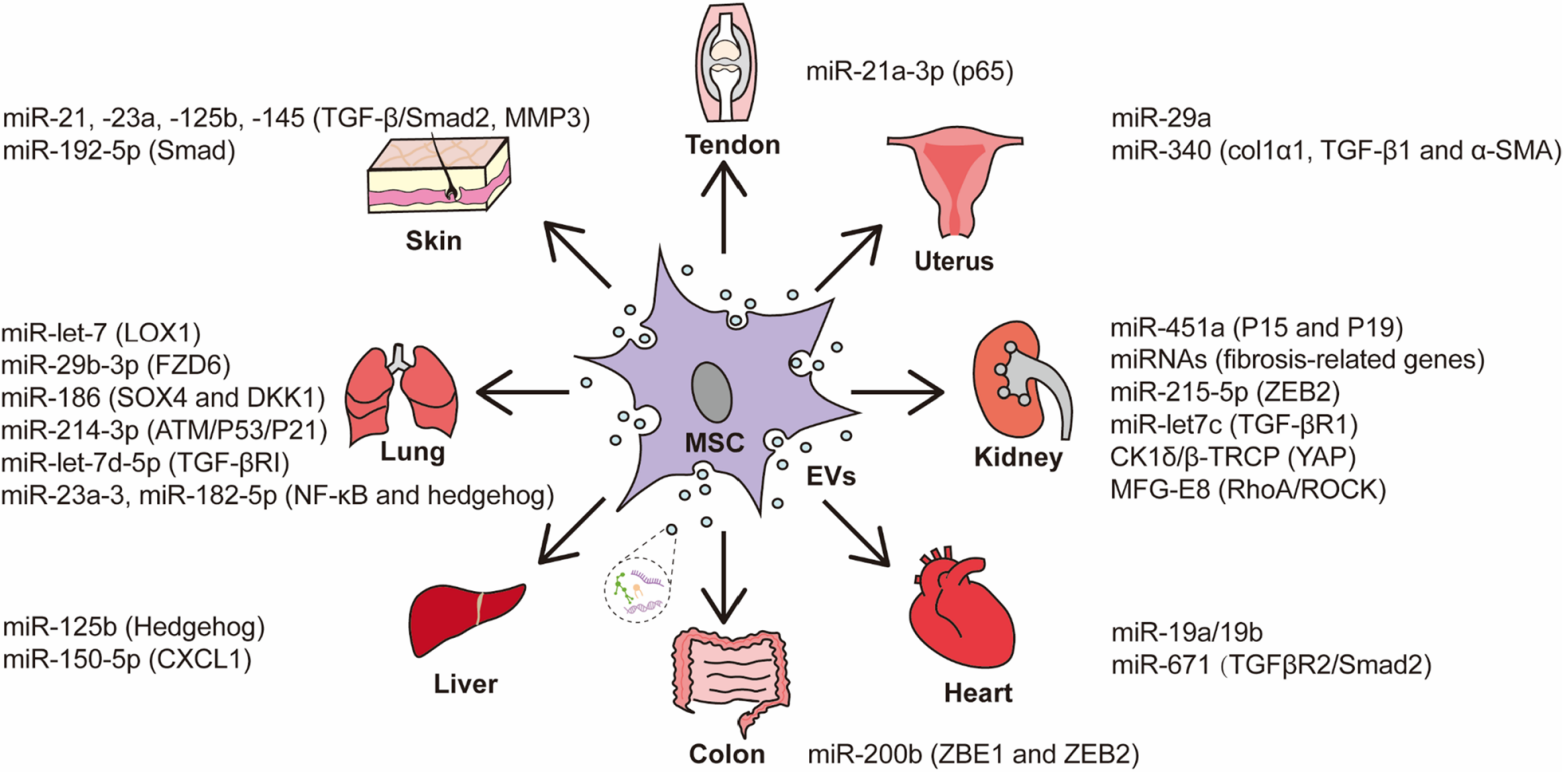
MSC-EVs, especially exosomes, can exert anti-fibrosis effect through all kinds of mechanisms. MSCs, mesenchymal stem cell; EVs, extracellular vesicles

MSC-EVs transplantation, as an emerging and novel therapy, has been confirmed the benefits for various diseases, including fibrotic diseases. A clinical trial (NCT04173650), *MSC EVs in Dystrophic Epidermolysis Bullosa*, estimated study start date in April 2021; however, current research on fibrosis is limited to the preclinical stage. Many preclinical trials established different fibrotic models (Table 1). Due to various reasons leading to fibrosis, there are many methods to induce fibrosis models, such as chemical poisons. Cells treating with TGF-β is a common way to induce EMT and fibrosis in most vitro trials. These preclinical studies have verified different MSC-EVs, especially exosomes, can exert anti-fibrosis effect through all kinds of mechanisms to treat fibrotic diseases. Additionally, miR-21/-23/-29/-let7 in MSC-EVs and TGFβ/Smad signalling pathway are common molecular mechanism in treating fibrotic diseases, such as fibrosis of lung, liver, kidney, skin, and heart (Fig. 2).

**Table 1:** Anti-fibrotic effect of MSC-EVs in different models

Organ	Model	Animal	In vitro model	Administration	Dosage	Source of EVs/Exos/MVs	Cargo in EVs	Mechanism	Effect	Refs.
Lung	BLM	C57BL/6 J mice	MLE-12 cells with TGF-β1	Tail vein	0.5 mg/kg/day for 7 days	MenSC-Exos	miR-let-7	↓LOX1	Regulating ROS, mtDNA damage, and NLRP3 inflammasome activation	[12]
	BLM	C57BL/6 mice	LL29	Tail vein	100 μg	BMMSC–EVs	miR-29b-3p	↓FZD6	Inhibiting fibroblast proliferation, migration, and differentiation	[13]
	BLM	C57BL/6 mice	LL29	Tail vein	100 μg	BMMSC–EVs	miR-186	↓SOX4 and DKK1	Restraining fibroblast activation	[14]
	BLM	C57BL/6 mice		Tail vein	8.6 × 10^8^ particles	BMMSC–EVs			Modulating monocyte phenotypes	[15]
	Radiotherapy	C57 mice		Tail vein	100 μg	hpMSC-EVs	miR-214-3p	↓ATM/P53/P21		[16]
	Pulmonary artery hypertension	Wistar rats	Pulmonary artery endothelial cells	Tail vein	25 µg for 3 days	huMSC-Exos		Wnt5a/BMP	Inhibiting EndMT	[17]
	PM2.5	SD rats	Type II alveolar epithelial cells	Intratracheal instillation	2.5 ~ 2.8 × 10^10^ particles	ADMSC-EVs	miR-let-7d-5p	↓TGF-βRI		[18]
	Lipopolysaccharide	C57BL/6 mice	MLE-12 cells	Tail vein	70 μg	BMMSC-Exos	miR-23a-3, miR-182-5p	↓NF-κB and hedgehog pathways via silencing Ikbkb and Usp5	Reversing EMT	[19]
Liver	CCl4	Mice	HL7702 with TGF-β1	Right lobes of livers	250 mg	huMSC-Exos		↓TGF-β1/Smad signalling pathway	Inhibiting EMT	[20]
	CCl4	SD rats	Human HSCs line LX2			CPMSC-Exos	miR-125b	↓Hedgehog signalling	Suppressing activation of HSCs	[21]
	CCl4	C57BL/6 J mice	HSCs with TGF-β	Tail vein		ADMSC-EVs	miR-150-5p	↓CXCL1		[22]
	CCl4	SD rats	HSCs	Tail vein	250 mg	BMMSC-Exos		↓Wnt/β-catenin signalling pathway	Inhibition of HSCs	[23]
	NASH; CCl4	SD rats	HSCs and KCs	Intravenous	15/20 μg/kg	AMSC-EVs		↓LPS/TLR4 signalling pathway	↓Activation of HSCs and Kupffer cells	[24]
	NASH	SCID mice		Tail vein	2.5 × 10^8^ particles	HLSC-EVs	251 proteins		↓Inflammation and cytokine pathways	[25]
Kidney	I/R	C57BL/6 mice	mTECs	Tail vein	100ug	ADMSC-Exos		↑Sox9		[26]
	STZ	Babl/c mice	HK-2	Tail vein	1.5 mg/kg	huMSC-MVs	miR-451a	↓P15 and P19	Inhibiting EMT	[27]
	STZ	NSG mice		Intravenous	1 × 10^10^ particles	HLSC/BMMSC-EVs	miRNAs	Fibrosis-related genes		[28]
	Aristolochic acid	NSG mice	mTECs	Intravenous	1 × 10^10^ particles	BMMSC-EVs		↓α-SMA, TGF-β1 and Col1a1 genes		[29]
	High glucose		MPC5 cells			ADMSC-Exos	miR-215-5p	↓ZEB2	Reversing EMT	[30]
	UUO	C57BL/6 J mice	NRK52E			BMMSC-Exos	miR-let7c	↓TGF-βR1		[31]
	UUO	SD rats		Tail vein	10 mg/kg	huMSC-Exos	CK1δ/β-TRCP	↓YAP		[32]
	UUO	SD rats	NRK-52E cells with TGF-β1	Renal artery	200 μg	huMSC-EVs		↓ROS-mediated P38MAPK/ERK signalling pathway		[33]
	UUO	SD rats	HK-2 cells with TGF-β1	Intravenous	0.5 mg/kg	BMMSC-EVs	MFG-E8	↓RhoA/ROCK signalling		[34]
Heart	TAC	C57BL/6 mice	NRVCs with AngII	Intramyocardial	20 μL	BMMSC-Exos			↑Senescence of myofibroblasts	[35]
	MI	Mice	HL-1 cardiac muscle cells		0.5 μmol	BMMSC-Exos	miR-19a/19b			[36]
	MI	Rats	H9c2 cells with hypoxia	Inferior vena cava	2.5 × 10^12^ particles	ADMSC-Exos		↑S1P/SK1/S1PR1 signalling		[37]
	MI	C57BL/6JNifdc mice	Cardiomyocytes with OGD	Boundary area of the infarcted cardiac	100 μg	ADMSC-Exos	miR-671	TGFβR2/Smad2		[38]
Skin	Full-thickness skin defect	ICR and nude mice	Fibroblasts with TGF-β	Inject around the wound	100 mg/ml	huMSC-Exos	miR-21, -23a, -125b, -145	↓TGF-β/Smad2 pathway	↓Myofibroblast differentiation	[39]
	Full-thickness skin defect	BALB/c mice	Dermal fibroblasts	Intravenous	200 μL	ADMSC-Exos		↑MMP3 expression via ERK/MAPK pathway	Regulating ratios of type III: type I collagens, TGF-β3: TGF-β1, and MMP3:TIMP1 and fibroblast differentiation	[40]
	Full-thickness skin defect	C57BL/6 mice		Intradermal	10 μg	MenSC-Exos			↓Col1: Col3 ratio	[41]
	Full-thickness skin defect	BALB/c mice	HSFs	Subcutaneous	70 μg	ADMSC-Exos	miR-192-5p /	Regulating Smad signalling pathway via IL-17RA	↓The proliferation and migration of HSFs, decreased collagen deposition	[42]
	Sclerodermatous cGVHD	BALB/c mice		Intraperitoneal	100 μg	huMSC-EVs		↓TGF-β/smad2	↓The activation of macrophages and B cells immune response	[43]
Uterus	IUA	ICR mice	Endometrial epithelial cells	Uterine cavity	100μL	BMMSC-Exos	miR-29a		↑Endometrial repair	[44]
	IUA	SD rats	Endometrial stromal cells	Tail vein		BMMSC-Exos	miR-340	↓col1α1, TGF-β1 and α-SMA expression		[45]
	IUA	Rabbits	Endometrial epithelial cells with TGF-β1	Muscle walls of the uterus	50 μg	BMMSC-Exos		↓TGF-β1/Smad pathway	Reverse EMT	[46]
Tendon	Tendon adhesion	SD rats	Fibroblast cells with TGF-β1	Subcutaneous	200 μg	huMSC-Exos	miR-21a-3p	↓p65		[47]
Colon	TNBS	SD rats	IEC-6 Cells with TGF-β1	Intravenous	10 μg/day for 6 days	BMMSC-MVs	miR-200b	↓ZBE1 and ZEB2	Inhibiting EMT	[48]

MSCs, mesenchymal stem cells; EVs, extracellular vesicles; MSC-EVs, mesenchymal stem cell-derived extracellular vesicles; Exos, exosomes; MVs, microvesicles; BLM, Bleomycin; EndMT, Endothelial-mesenchymal transition; EMT, epithelial mesenchymal transition; CCl4, carbon tetrachloride; HSCs, hepatic stellate cells; NASH, nonalcoholic steatohepatitis; I/R, ischemia/reperfusion; STZ, streptozotocin; UUO, unilateral ureteral obstruction; TAC, transverse aortic constriction; MI, myocardial infarction; HSFs, hypertrophic scar-derived fibroblasts; IUA, intrauterine adhesion

### MSC-EVs and pulmonary fibrosis

Pulmonary fibrosis (PF) is considered as a common manifestation of multiple lung diseases, such as some chronic diseases including IPF, silicosis, and pulmonary arterial hypertension [49]. Owing to the multiple underlying pathogenesis of PF and the lack of effective therapeutic drugs available clinically, developing new methods and drugs for preventing and treating PF has become the focus of many studies. MSCs have been verified to be effective in alleviating PF in clinical trials. Zhang et al. reported that an IPF patient subjected to long-term oxygen therapy exhibited improvements in physical performance, quality of life, and respiratory parameters after receiving huMSCs intravenous infusion over a follow-up period of 12 months [50]. PF is generally induced by intratracheal instillation of Bleomycin (BLM) in preclinical trials.

It should be pointed out that MSC-EVs as a potential antiviral therapy was reported in COVID-19 disease. Several clinical trials (NCT04276987, NCT04491240, and NCT04493242) are exploring the use of MSC-EVs in treating COVID-19 disease. Gentile et al. reviewed that intravenous infusion of MSCs played a significant role in counteracting fibrosis in individuals infected with SARS-CoV-2 by the secreted EVs, especially exosomes [51]. In addition, ADMSCs and Stromal Vascular Fraction Cells (SVFs) improved lung fibrosis of COVID-19 patients through exosomal miRNAs [52].

The anti-fibrosis effect of miRNAs in MSC-EVs has become a research hotspot for PF induced by BLM. Sun et al. showed that exosomal miR-let-7 from MenSCs alleviates PF by regulating reactive oxygen species, mitochondrial DNA damage, and NLRP3 inflammasome activation [12]. Fibroblast proliferation is key to fibrosis. Wan et al. reported that BMMSC-EVs suppress fibroblast proliferation by down-regulating FZD6 expression in fibroblasts via miR-29b-3p [13]. Similarly, Zhou et al. found that miR-186 in BMMSC-EVs alleviates IPF by suppressing the expression of SOX4 and DKK1, thus blocking fibroblast activation [14]. Certainly, fibrosis is also closely correlated with inflammation. Mansouri et al. experimentally demonstrated that MSC-Exos prevent and reverse experimental PF by systematically modulating monocyte phenotypes [15].

MSC-EVs also ameliorate PF in other PF models. Chaubey et al. considered that TNF α-stimulated gene-6 (TSG-6) in huMSC-Exos play an important role in the hyperoxia-induced bronchopulmonary dysplasia model [53]. As for PF induced by intratracheal instillation of silica, the fibrosis gene, and inflammation are preliminarily explored, and mechanism still less studied. Phinney et al. further revealed that BMMSC-Exos reduce the extent of infiltration of monocytes and expression of profibrogenic genes (IL-10, and Col1α1) in the lung, thus relieving PF [54]. MiR-214-3p in hpMSC-EVs attenuates radiation-induced DNA damage by down-regulating the ATM/P53/P21 signalling pathway in PF induced by radiotherapy of the lung with a malignant tumour, thus relieving pulmonary inflammation and fibrosis [16]. Zhang et al. showed that huMSC-Exos block the progress of fibrosis by regulating the Wnt5a/BMP signalling pathway in pulmonary arterial hypertension models in vivo and in vitro [17]. Gao et al. found that ADMSC-EVs inhibit TGF-βRI by transferring miR-let-7d-5p, further relieving PM2.5-induced lung injury and PF [18]. Xiao et al. recently found that miR-23a-3 and miR-182-5p in BMMSC-Exos reverse the EMT process by blocking the activation of NF-κB and hedgehog pathways via silencing of Ikbkb and destabilising IKKβ in lipopolysaccharide (LPS)-induced lung injury [19].

### MSC-EVs and liver fibrosis

Liver fibrosis is triggered by chronic injury and inflammation, leading to liver cirrhosis, portal hypertension, and liver failure. At present, the therapy on liver cirrhosis with MSCs has realised remarkable achievements in clinical trials. A pilot four-phase clinical trial (NCT04243681) regarding MSCs infusion for treating decompensated cirrhosis has been completed in India. Numerous studies have been conducted on preclinical therapy of liver fibrosis with MSC-EVs.

It is common to induce liver fibrosis by using carbon tetrachloride (CCl_4_) during preclinical trials. Li et al. suggested that huMSC-Exos alleviate liver fibrosis by inactivating the TGF-β1/Smad signalling pathway and inhibiting EMT [20]. Hyun et al. showed that miR-125b in exosomes of chorionic plate-derived mesenchymal stem cells (CP-MSCs) drives the regression of fibrosis by inhibiting the activation of hedgehog signals [21]. Additionally, Du et al. proposed that miR-150-5p in ADMSC-EVs can weaken CCl_4_-induced liver fibrosis by inhibiting the expression of CXCL1 [22]. Ohara et al. reported that EVs from amnion-derived MSCs (AMSC-EVs) ameliorate liver fibrosis by weakening the activation of hepatic stellate cells and Kupffer cells [24]. Rong et al. further proved that BMMSCs-Exos can alleviate CCL_4_-induced liver fibrosis by inhibiting the activation of hepatic stellate cells via the Wnt/β-catenin pathway [23].MSC-EVs can also improve liver function to some extent in the other models for liver fibrosis. For example, Bruno et al. found that HLSC-derived EVs attenuate liver fibrosis and inflammation by regulating fibrosis genes in a murine model of non-alcoholic steatohepatitis (NASH) [25].

Exogenous modification, preconditioning, and the use of bio-gel prove to be effective in strengthening the anti-fibrosis effect of MSC-EVs in liver. MiR-122 modification can improve the therapeutic efficacy of ADMSCs via exosome-mediated miR-122 communication [55]. The miR-181-5p-bearing exosomes increase autophagy and reduce TGF-β1-induced liver fibrosis by inhibiting the STAT3/Bcl-2/Beclin1 pathway [56]. Similarly, exosomes derived from mmu_circ_0000623-modified ADMSCs prevent liver fibrosis by activating autophagy [57]. The exosomes derived from miR-145-5p-modified huMSCs alleviate liver fibrosis by down-regulating the expression of actin-binding protein 1 [7]. The PEG hydrogels can prolong the bioavailability of MSC-EVs in targeted livers, thus enhancing the anti-fibrosis characteristics thereof [58]. Relative to MSC-EVs, MSC-EVs preconditioned with IFN-γ better alleviate the inflammation and fibrosis of the murine model with liver cirrhosis [59].

### MSC-EVs and renal fibrosis

Renal fibrosis is mainly induced by various chronic kidney diseases. It is considered the main pathological change and common pathway in the final stage of kidney disease, however, no targeted therapy yet exists to reverse renal fibrosis [60]. At present, research further verifies that MSC-EVs are the key to treating renal fibrosis models.

Multiple studies have shown that MSC-EVs can relieve renal fibrosis. MSC-EVs can inhibit the fibrotic transformation of various renal parenchyma cells at a cellular level. Zhu et al. believed that ADMSC-Exos block the transition of TGF-β1-induced tubular epithelial cells to their profibrogenic phenotype [26]. Zhong et al. verified that huMSC-MVs reboots the cell cycle and reverses the EMT in vivo and in vitro through negative regulation of P15 and P19 via miR-451a [27]. Jin et al. found that ADMSC-Exos attenuate the EMT of podocytes by inhibiting ZEB2 via miR-215-5p [30]. MSC-EVs slow the progress of renal fibrosis by affecting fibrosis-related genes at a molecular level. Wang et al. showed that BMMSCs can suppress the expression of fibrosis genes by delivering exogenous miR-let-7c via exosomes [31]. Grange et al. revealed that MSC-EVs down-regulate fibrosis genes in a chronic renal injury model induced by diabetes mellitus [28]. Kholia et al. reported that MSC-EVs significantly down-regulate the fibrosis genes α-SMA, TGF-β1, and Col1a1 after their having been up-regulated by aristolochic acid [29]. In addition, MSC-EVs also alleviate renal fibrosis via various signalling pathways: Ji et al. found that huMSC-Exos attenuate renal fibrosis through CK1δ/β-TRCP-mediated YAP degradation in a UUO model [32]. Similarly, Liu et al. suggested that huMSC-Exos protect against renal interstitial fibrosis through the ROS-mediated P38MAPK/ERK signalling pathway [33]. Shi et al. found that BMMSC-EVs attenuate renal fibrosis, in part by inhibiting the RhoA/ROCK pathway [34].

Researchers have developed many methods with which to enhance the characteristics of MSC-EVs against renal fibrosis. Modification of MSC-EVs is taken as an approach to strengthen the anti-fibrotic nature thereof. Zhou et al. modified EVs via KMP2 to ameliorate chronic renal fibrosis in I/R mice [61]. Chen et al. suggested that GDNF-modified ADMSC-Exos alleviate renal fibrosis by activating the SIRT1/eNOS signalling pathway [62]. Also, Zhang et al. prolonged the release of EVs through use of RGD hydrogels [63]. The collagen matrices employed by Liu et al. exhibit similar characteristics [64]. Additionally, Zhang et al. revealed that the over-expression of Oct-4 enhances the anti-fibrotic effect of MSC-EVs [65].

### MSC-EVs and cardiac fibrosis

Myocardial infarction (MI) is regarded as one of the main causes of death from cardiovascular disease. The damaged cardiomyocytes are replaced with fibrous scars and cardiac remodelling leads to cardiac dysfunction [66]. Myocardial remodelling has long been a focus of research (post-MI). The amelioration of myocardial fibrosis with MSC-EVs has become a research hotspot.

Cardiac fibroblasts are crucial for myocardial fibrosis. Chen et al. found that BMMSC-Exos promotes the early senescence of myofibroblasts in vitro [35]. Ferguson et al. reported that MSC-Exos can inhibit the generation of type I collagen genes in primary human cardiac fibroblasts [67]. Additionally, MSC-EVs relieve cardiac remodelling by delivering diverse information. Wang et al. suggested that BMMSC-Exos can promote recovery of heart function via miR-19a/19b to decrease myocardial fibrosis in a MI model [36]. Deng et al. suggested that ADMSC-Exos alleviates myocardial injuries after MI by activating the S1P/SK1/S1PR1 signalling pathway and promoting macrophage M2 polarisation [37]. Recently, Wang et al. found that miR-671 in ADMSC-Exos alleviate myocardial injury via targeting TGFβR2/Smad2 axis in vivo and vitro [38].

Like liver, kidney, and other organs, many methods can assist MSC-EVs in improving cardiac function and alleviating fibrosis, as shown in Table 2. MSC-EVs are parcelled in PGN hydrogels [68], alginate hydrogels [69], and (RADA)_4_-SDKP hydrogels [70] to prolong retention so that EVs can be stably and sustainably released. The modification and preconditioning of EVs also can reduce cardiac fibrosis. Pan et al. revealed that miR-146a-modified ADMSC-Exos inhibit the expression of EGR1 after transcription, which reverses acute myocardial infarction or hypoxia-induced TLR4/NFκB signal activation. The activation of TLR4/NFκB signals plays an important role in promoting cardiomyocyte apoptosis, inflammatory response, and fibrosis [71]. The exosomes engineered from IMTP can specifically target the ischemic myocardium [72]. FNDC5 or irisin increases the secretion of exosomes from BMMSCs [73]. Those exosomes with over-expressed HIF-1α protect cardiac function by promoting neovascularisation and inhibiting fibrosis [74]. B2M-deficient huMSC-Exos inhibit cardiac fibrosis and restores cardiac function [75]. The ischemically preconditioned exosomes relieve fibrosis after MI through miR-22 [76]. Hypoxia strengthens the activities of miR-210 and neutral sphingomyelinase 2 in MSC-Exos, thus playing a cardioprotective role [77].

**Table 2:** Methods to enhance the anti-fibrosis effect of MSC-EVs in different organs

	Exogenous modification	Bio-gel	Preconditioning
Liver	miR-122 [55]	PEG hydrogels [58]	IFN-γ [59]
	miR-181-5p [56]		
	mmu_circ_0000623 [57]		
	miR-145-5p [7]		
Kidney	KMP2 [61]	RGD hydrogels [63]	Oct-4 overexpression [65]
	GDNF [62]	Collagen matrix [64]	
Heart	miR-146a [71]	PGN hydrogels [68]	FNDC5 or irisin [73]
	IMTP [72]	Alginate hydrogels [69]	HIF-1α [74]
		(RADA)_4_-SDKP hydrogels [70]	B2M-deficient [75]
			Ischemia [76]
			Hypoxia [77]
Skin	TSG-6 [78]	BSSPD hydrogels [79]	
Tendon adhesion			Hydroxycamptothecin [80]
Urethra			TNFα [81]

MSCs, mesenchymal stem cells; MSC-EVs, mesenchymal stem cell-derived extracellular vesicles

### MSC-EVs and skin scars

Skin scars are common and an effective therapeutic method for human scarless wound-healing remains to be determined. Fang et al. suggested that huMSC-Exos suppress myofibroblast differentiation by inhibiting the TGF-β/Smad2 pathway during the wound-healing [39]. Wang et al. found that ADMSC-Exos reduce scar-formation by regulating the ratios of type III: type I collagens, TGF-β3: TGF-β1, and MMP3: TIMP1 and fibroblast differentiation [40]. Dalirfardouei et al. thought that MenSC-Exos may alleviate scar formation by reducing the Col1: Col3 ratio [41]. ADMSC-Exos ameliorate fibrosis via the miR-192-5p/IL-17RA/Smad axis in hypertrophic scars [42]. HuMSC-EVs prevent fibrosis in the cGVHD mouse model with scleroderma by suppressing the activation of macrophages and B cell immune response [43]. Jiang et al. suggested that TSG-6-modified MSC-Exos attenuate collagen deposition and scar-formation during the wound-healing [78]. Additionally, miR-29b-3p secreted by BMMSC-EVs parcelled in BSSPD hydrogels inhibits the proliferation and migration of endothelial cells and fibroblasts and the expression of Col1A1 of fibroblasts by curbing the PI3K/Akt, Erk1/2, and Smad3/TGF-β1 signalling pathways, thus realising scarless wound-healing [79].

### MSC-EVs and other fibrotic diseases

Intrauterine adhesion (IUA), also called Asherman syndrome, triggered by endometrial injury is a common gynaecological disease induced by basement membrane injury in the endometrium and exposure of myometrial tissues. At present, IUA treatment mainly relies on hysteroscopic surgery to eliminate adhesions, in concert with hormonotherapy [82]. Tan, Xia, and Ying thought that miR-29a in BMMSCs-Exos may be an important factor affecting resistance to fibrosis during endometrial repair of IUA [44]. Xiao et al. found that BMMSC-Exos alleviate endometrial fibrosis by transferring miR-340 to endometrial stromal cells [45]. In addition, Yao et al. verified that BMMSC-Exos can reverse the EMT of rabbit endometrial epithelial cells through the TGF-β1/Smad pathway [46]. Tendon adhesion, a common complication of tendon injuries, influences the recovery of motor function. Yao et al. showed that huMSC-Exos are likely to regulate the p65 activity by delivering low-abundance miR-21a-3p, while showing anti-adhesion characteristics [47]. Li et al. revealed that hydroxycamptothecin-induced huMSC-EVs inhibit myofibroblast differentiation by activating the endoplasmic reticulum of fibroblasts and exhibit a strengthened anti-adhesion effect after Achilles tendon injury [80]. Moreover, exosomes can prevent the formation of urethral stricture by regulating fibrosis and angiogenesis, TNFα-preconditioned huMSC-Exos are more effective in inhibiting urethra fibrosis and stricture via up-regulating miR-146a compared to untreated huMSC-Exos [81]. BMMSC-MVs can inhibit the EMT and further colonic fibrosis by targeting ZEB1 and ZEB2 in vivo and in vitro through miR-200b [48].

## Problems and prospects

MSC-EVs exert a positive effect on various fibrotic diseases in the aforementioned research. The current research aiming at treatment of fibrosis with MSC-EVs is in its preclinical stage. There was a lack of large animal experiments in MSC-EVs treating fibrotic diseases; however, several studies all found that MSC-EVs can attenuate renal or myocardial fibrosis in the swine models of metabolic syndrome and renovascular disease by restoring the renal microcirculation and preserving renal cellular integrity [83–85]. Even in research on the treatment of peritoneal dialysis, MSCs for peritoneal fibrosis remain under exploration and MSC-EVs have rarely been involved in the research. Moreover, huMSCs relieve peritoneal fibrosis in rats induced by MGO by up-regulating miR-153-3p [86]. Numerous tests are still needed to support further clinical research in MSCs. Most fibrosis models have not been normalised for different tissues and organs. PF is mainly induced by using BLM and silica and the liver fibrosis is mainly triggered with CCl_4_ in animal experiments; it is possible to induce fibrosis of different tissues and organs with TGF-β1 during the cell tests.

The dosage and administration route of MSC-EVs are still under exploration and the most effective administration has not yet been determined. A systematic review concluded that localised administration for delivering MSC-EVs was common in treating ophthalmic, skin and musculoskeletal conditions, whereas systemic administration was popular in autoimmune, brain, gastrointestinal, liver and pancreatic diseases [87]. Similar to MSCs, topical application of MSC-EVs is the least invasive method; arterial injection and direct injection can reduce EVs loss; intravenous administration is relatively ease and safe [88].

Overall, this review summarises preclinical studies on application of MSC-EVs in treating various fibrotic diseases, including lung, liver, kidney, heart, skin, and endometrium. MSC-EVs are promising as drugs for treating fibrotic diseases in preclinical trials. More efforts are needed before further clinical application due to the limited research completed at time of writing.

## Data Availability

Not applicable.

## Abbreviations

MSCs: Mesenchymal stem cells
MSC-EVs: Mesenchymal stem cell-derived extracellular vesicles
IPF: Idiopathic pulmonary fibrosis
BMMSCs: Bone marrow-derived MSCs
ADMSCs: Adipose-derived MSCs
huMSCs: Human umbilical cord-derived MSCs
hpMSCs: Human placenta-derived MSCs
HLSCs: Human liver stem cells
MenSCs: Menstrual blood-derived stem cells
MVs: Microvesicles
Exos: Exosomes
ADMSC-EVs: EVs from ADMSCs
EMT: Epithelial mesenchymal transition
PF: Pulmonary fibrosis
BLM: Bleomycin
SVFs: Stromal Vascular Fraction Cells
MSC-CM: Conditioned medium of MSCs
HAECs: Human amniotic epithelial cells
TSG-6: TNF α-stimulated gene-6
CCl_4_: Carbon tetrachloride
CP-MSCs: Chorionic plate-derived mesenchymal stem cells
AMSC-EVs: EVs from amnion-derived MSCs
NASH: Non-alcoholic steatohepatitis
UUO: Unilateral ureteral obstruction
MI: Myocardial infarction
IUA: Intrauterine adhesion
